# Long-term efficacy and safety of first-line ibrutinib treatment for patients with CLL/SLL: 5 years of follow-up from the phase 3 RESONATE-2 study

**DOI:** 10.1038/s41375-019-0602-x

**Published:** 2019-10-18

**Authors:** Jan A. Burger, Paul M. Barr, Tadeusz Robak, Carolyn Owen, Paolo Ghia, Alessandra Tedeschi, Osnat Bairey, Peter Hillmen, Steven E. Coutre, Stephen Devereux, Sebastian Grosicki, Helen McCarthy, David Simpson, Fritz Offner, Carol Moreno, Sandra Dai, Indu Lal, James P. Dean, Thomas J. Kipps

**Affiliations:** 1 Department of Leukemia, University of Texas MD Anderson Cancer Center, Houston, TX USA; 2 Wilmot Cancer Institute, University of Rochester Medical Center, Rochester, NY USA; 3 Medical University of Lodz, Copernicus Memorial Hospital, Lodz, Poland; 4 Tom Baker Cancer Centre, University of Calgary, Calgary, AB Canada; 5 Università Vita-Salute San Raffaele and IRCCS Ospedale San Raffaele, Milan, Italy; 6 ASST Grande Ospedale Metropolitano Niguarda, Milan, Italy; 7 Rabin Medical Center, Petah Tikva, Israel and Sackler Faculty of Medicine, Tel Aviv University, Tel Aviv, Israel; 8 The Leeds Teaching Hospitals, St. James Institute of Oncology, University of Leeds, Leeds, UK; 9 Stanford Cancer Center, Stanford University School of Medicine, Stanford, CA USA; 10 Kings College Hospital, NHS Foundation Trust, London, UK; 11 Department of Hematology and Cancer Prevention, Silesiam Medical University, Katowice, Poland; 12 Royal Bournemouth General Hospital, Bournemouth, UK; 13 North Shore Hospital, Auckland, New Zealand; 14 Universitair Ziekenhuis Gent, Gent, Belgium; 15 Hospital de la Santa Creu i Sant Pau, Autonomous University of Barcelona, Barcelona, Spain; 16 Pharmacyclics LLC, an AbbVie Company, Sunnyvale, CA USA; 17 UCSD Moores Cancer Center, San Diego, CA USA

**Keywords:** Chronic lymphocytic leukaemia, Targeted therapies

## Abstract

RESONATE-2 is a phase 3 study of first-line ibrutinib versus chlorambucil in chronic lymphocytic leukemia (CLL)/small lymphocytic lymphoma (SLL). Patients aged ≥65 years (*n* = 269) were randomized 1:1 to once-daily ibrutinib 420 mg continuously or chlorambucil 0.5–0.8 mg/kg for ≤12 cycles. With a median (range) follow-up of 60 months (0.1–66), progression-free survival (PFS) and overall survival (OS) benefits for ibrutinib versus chlorambucil were sustained (PFS estimates at 5 years: 70% vs 12%; HR [95% CI]: 0.146 [0.098–0.218]; OS estimates at 5 years: 83% vs 68%; HR [95% CI]: 0.450 [0.266–0.761]). Ibrutinib benefit was also consistent in patients with high prognostic risk (*TP53* mutation, 11q deletion, and/or unmutated IGHV*)* (PFS: HR [95% CI]: 0.083 [0.047–0.145]; OS: HR [95% CI]: 0.366 [0.181–0.736]). Investigator-assessed overall response rate was 92% with ibrutinib (complete response, 30%; 11% at primary analysis). Common grade ≥3 adverse events (AEs) included neutropenia (13%), pneumonia (12%), hypertension (8%), anemia (7%), and hyponatremia (6%); occurrence of most events as well as discontinuations due to AEs decreased over time. Fifty-eight percent of patients continue to receive ibrutinib. Single-agent ibrutinib demonstrated sustained PFS and OS benefit versus chlorambucil and increased depth of response over time.

## Introduction

Chronic lymphocytic leukemia (CLL) predominantly affects older individuals who frequently have comorbidities that may preclude the use of intensive chemoimmunotherapy regimens, such as fludarabine, cyclophosphamide, and rituximab (FCR) [1–3]. Before establishment of the chlorambucil-based CD20 combinations, single-agent chlorambucil was considered a standard of care in older patients with CLL [4, 5].

Ibrutinib is a first-in-class, oral, once-daily inhibitor of Bruton’s tyrosine kinase (BTK), which as a single agent has led to prolonged progression-free survival (PFS) and overall survival (OS) in patients with previously treated CLL [6, 7]. RESONATE-2 is an international phase 3 study evaluating the efficacy and safety of first-line ibrutinib compared with chlorambucil in older patients with CLL or small lymphocytic lymphoma (SLL) [8]. The primary analysis (median follow-up of 18.4 months) demonstrated an 84% reduction in the risk of disease progression (PD) or death (as assessed by an independent review committee) and significant improvement in OS for ibrutinib compared with chlorambucil, supporting the initial approval of first-line ibrutinib for CLL/SLL in the United States and for CLL in the European Union, with ibrutinib now approved for CLL in over 90 countries [8–10]. Data previously reported from this study after a median follow-up of 28.5 months demonstrated a sustained PFS benefit for ibrutinib and improved depth of response over time, with no new unexpected safety concerns [11]. Continued long-term study follow-up is important to provide quantitative assessments of response durability, dimensions of patients’ well-being, and safety with continuous single-agent ibrutinib treatment to inform clinical practice.

Herein, we present the efficacy and safety outcomes for first-line ibrutinib treatment after a median follow-up of 5 years from the RESONATE-2 study. This represents the longest follow-up to date from a phase 3 trial of BTK-directed therapy in the first-line setting for CLL.

## Subjects and methods

### Study design and population

RESONATE-2 collectively includes the phase 3, open-label, international, randomized study PCYC-1115 and extension study (PCYC-1116) comparing the efficacy and safety of ibrutinib versus chlorambucil in first-line CLL/SLL. Detailed methods have been previously reported [8]. Briefly, previously untreated patients without chromosome 17p deletion [del(17p)] aged ≥65 years with CLL/SLL requiring therapy per published criteria [12] were randomized in a 1:1 ratio to oral ibrutinib (420 mg once daily) until PD or unacceptable toxicity, or 12 cycles of chlorambucil (0.5 mg/kg, increased up to 0.8 mg/kg as tolerated, on days 1 and 15 of each 28-day cycle). Following confirmation of PD, patients randomized to chlorambucil were eligible to cross over to second-line treatment with ibrutinib.

This study was conducted according to principles of the Declaration of Helsinki and the International Conference on Harmonisation Guidelines for Good Clinical Practice and was approved by the institutional review boards of participating institutions. All patients provided written informed consent. This study was registered with ClinicalTrials.gov, numbers NCT01722487 and NCT01724346.

### Endpoints and assessments

Endpoints included PFS, OS, overall response rate (ORR), improvement in hematologic parameters, patient-reported outcomes, and safety. Long-term PD and response were assessed by the investigator per 2008 International Workshop on CLL (iwCLL) criteria [12]. Long-term safety data are reported for patients who were initially randomized to ibrutinib. Nonhematologic adverse events (AEs) were graded using Common Terminology Criteria for Adverse Events, v4.03 [13]. Hematologic AEs were graded using iwCLL criteria [12].

### Statistical analysis

PFS and OS were analyzed according to the Kaplan–Meier method. To adjust for the impact of crossover on OS, sensitivity analyses were performed as previously described [14].

### Data sharing statement

Requests for access to individual participant data from clinical studies conducted by Pharmacyclics LLC, an AbbVie Company, can be submitted through Yale Open Data Access Project site at http://yoda.yale.edu.

## Results

### Patients

A total of 269 patients were randomized to ibrutinib (*n* = 136) or chlorambucil (*n* = 133; Supplementary Fig. 1). As previously reported, baseline characteristics were well balanced across treatment arms (Table 1) [8]. Treatment with first-line ibrutinib was ongoing in 79 (58%) patients after a median follow-up of 60 months (range, 0.1–66 months), while 56 (41%) discontinued treatment (Table 2). Of 133 patients randomized to chlorambucil, 96 experienced PD (75 crossed over to ibrutinib and 21 did not cross over after PD), one crossed over to receive ibrutinib without documented PD, and 36 patients remained on the chlorambucil arm without PD. Of 21 patients who did not cross over after PD (cross over to ibrutinib was not mandatory), six died, six were still on study without crossing over, and six discontinued the study. Of the 36 patients remaining on the chlorambucil arm without PD, 16 were still on study, 11 discontinued the study, and nine died.

**Table 1:** Baseline characteristics

	Ibrutinib *n* = 136	Chlorambucil *n* = 133
Median age (range), years	73 (65–89)	72 (65–90)
≥70 years, *n* (%)	96 (71)	93 (70)
Male, *n* (%)	88 (65)	81 (61)
ECOG performance status, *n* (%)		
0	60 (44)	54 (41)
1–2	76 (56)	79 (59)
Rai stage III or IV, *n* (%)	60 (44)	62 (47)
CIRS score >6, *n* (%)	42 (31)	44 (33)
Creatinine clearance <60 mL/min, *n* (%)	60 (44)	67 (50)
Bulky disease ≥5 cm, *n* (%)	54 (40)	40 (30)
β2-microglobulin >3.5 mg/L, *n* (%)	85 (63)	89 (67)
Hemoglobin ≤11 g/dL, *n* (%)	51 (38)	55 (41)
Platelet count ≤100 × 10^9^/L, *n* (%)	35 (26)	28 (21)
del(11q), *n*/*N* (%)	29/130 (22)	25/121 (21)
Unmutated IGHV, *n*/*N* (%)	58/101 (57)	60/103 (58)
*TP53* mutation *n/N* (%)	12/124 (10)	3/94 (3)
High prognostic risk features,^a^ *n* (%)	74 (54)	69 (52)

*CIRS* Cumulative Illness Rating Scale, *ECOG* Eastern Cooperative Oncology Group, *IGHV* immunoglobulin heavy chain variable region

^a^*TP53* mutation, del(11q), and/or unmutated IGHV

**Table 2:** Duration of treatment with first-line ibrutinib

	Ibrutinib *n* = 136
Median (range) duration of ibrutinib treatment, months^a^	57.1 (0.7–66.0)
Treatment duration, *n* (%)	
>3 years	99 (73)
>4 years	88 (65)
>5 years	37 (27)
Continuing ibrutinib on study, *n* (%)	79 (58)
Continuing on commercial ibrutinib, *n* (%)	0 (0)
Discontinued ibrutinib, *n* (%)	56 (41)
Adverse event	29 (21)
Progressive disease	8 (6)
Death	8 (6)
Withdrawal by patient	7 (5)
Investigator decision	4 (3)

^a^One patient did not receive any doses of ibrutinib

### Progression-free survival and overall survival

**Fig. 1:**
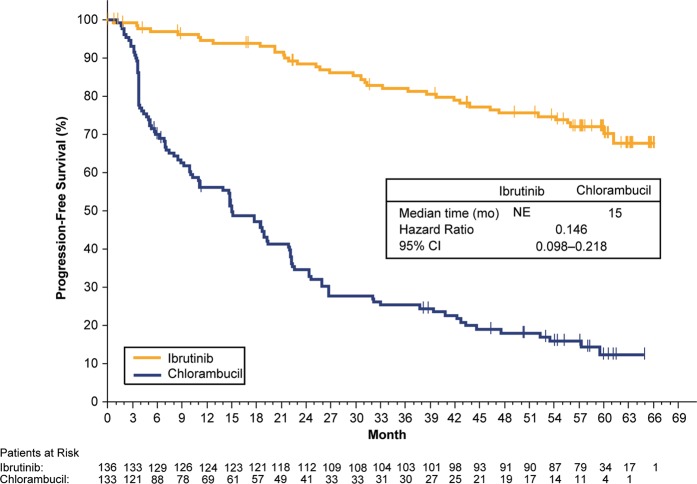
Progression-free survival with single-agent ibrutinib versus chlorambucil in first-line treatment for CLL/SLL. Survival analyses are from randomization until event or censored at last evidence of non-PD; vertical tick marks indicate censored patients. CI confidence interval, CLL chronic lymphocytic leukemia, NE not estimable, PD progressive disease, SLL small lymphocytic lymphoma

Ibrutinib significantly prolonged PFS compared with chlorambucil (median not reached vs 15.0 months [95% confidence interval (CI): 10.2–19.4]), with an 85% reduction in the risk of PD or death (hazard ratio, 0.146 [95% CI: 0.098–0.218]; Fig. 1). At 5 years, 70% of patients treated with ibrutinib and 12% with chlorambucil were estimated to be progression-free and alive. Eight (6%) of 136 patients discontinued ibrutinib due to PD.

**Fig. 2:**
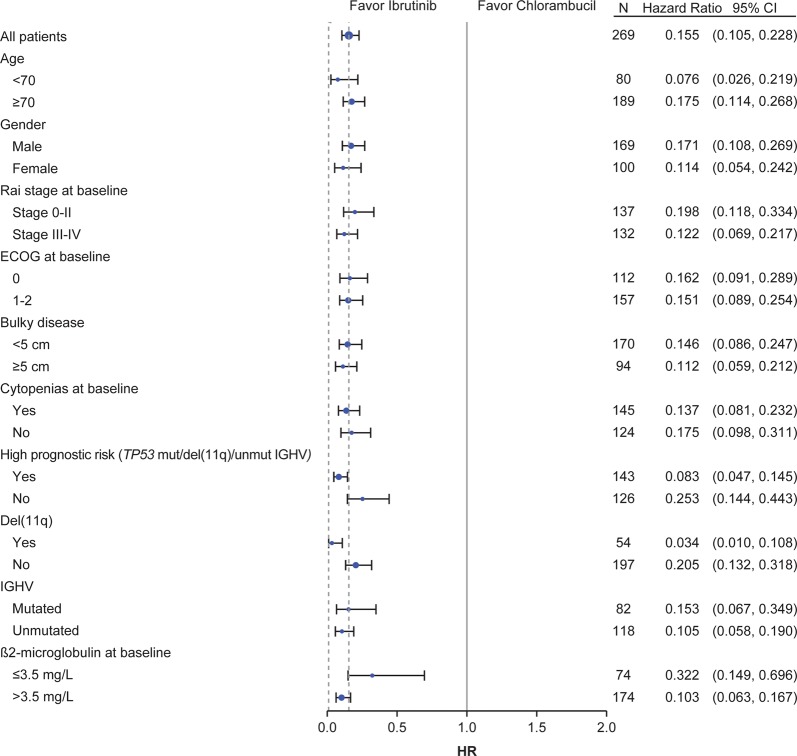
Progression-free survival according to baseline factor subgroups of interest. CI confidence interval, del(11q) chromosome 11q deletion, ECOG Eastern Cooperative Oncology Group, HR hazard ratio, IGHV immunoglobulin heavy chain variable region

**Fig. 3:**
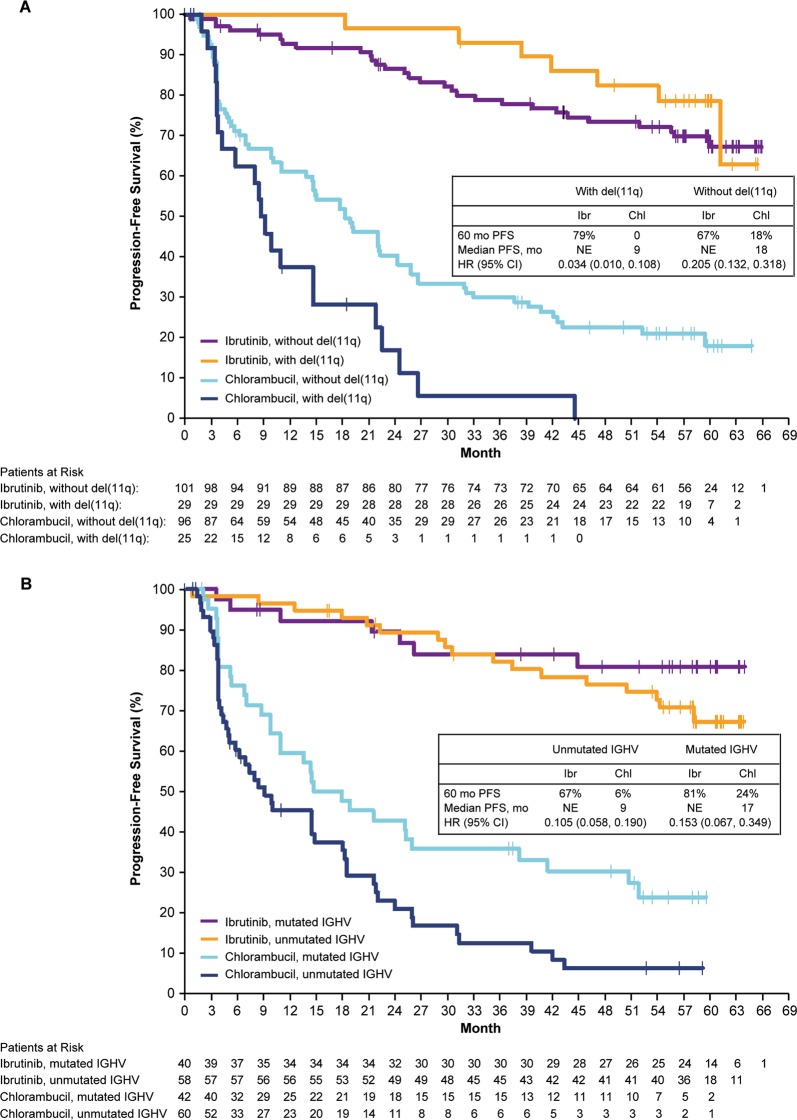
Progression-free survival by **a** del(11q) status and **b** IGHV mutational status. Survival analyses are from randomization until event or censored at last follow-up; vertical tick marks indicate censored patients. CI confidence interval, del(11q) chromosome 11q deletion, IGHV immunoglobulin heavy chain variable region, NE not estimable, NR not reached, PFS progression-free survival

A PFS benefit for ibrutinib over chlorambucil was observed across all patient subgroups examined, including those with the high-risk prognostic features of *TP53* mutation, chromosome 11q deletion (del[11q]), and/or unmutated immunoglobulin heavy chain variable region (IGHV) (PFS: HR 0.083 [95% CI: 0.047–0.145]) (Fig. 2). When examined individually, the presence of del(11q) and unmutated IGHV were each also associated with increased PFS in ibrutinib-treated patients compared with chlorambucil-treated patients. Ibrutinib dramatically reduced the risk of PD or death by 97% compared with chlorambucil in patients with del(11q) (Fig. 3a). Ibrutinib reduced the risk of PD or death by 90% and 85% compared with chlorambucil for patients with either unmutated and mutated IGHV, respectively (Fig. 3b). PFS was not significantly different for ibrutinib-treated patients with unmutated and mutated IGHV. In patients treated with ibrutinib, 79% of patients with del(11q) and 67% of patients with unmutated IGHV were estimated alive and progression-free at 5 years. Though patients with del(17p) CLL were excluded from the study, 12 ibrutinib-treated patients had *TP53* mutation; median PFS was not reached for ibrutinib-treated patients with *TP53* mutation or *TP53* wild type (HR [95% CI: 0.866 [0.264–2.846]) and the 5-year estimates were 56% and 73%, respectively. Only three patients randomized to chlorambucil had *TP53* mutation so no comparison could be made between treatments.

Median OS was not reached for either the ibrutinib or chlorambucil arms (HR [95% CI], 0.450 [0.266–0.761]). OS estimates at 5 years were 83% for ibrutinib and 68% for chlorambucil without censoring for crossover from chlorambucil to ibrutinib and were 80% for chlorambucil after censoring for crossover to ibrutinib treatment after PD (Supplementary Table 1). In patients with high prognostic risk CLL (*TP53* mutation, del[11q], and/or unmutated IGHV), OS at 5 years without censoring for crossover to ibrutinib was 84% for ibrutinib and 62% for chlorambucil (HR 0.376 [95% CI: 0.180–0.786]).

### Overall response

**Fig. 4:**
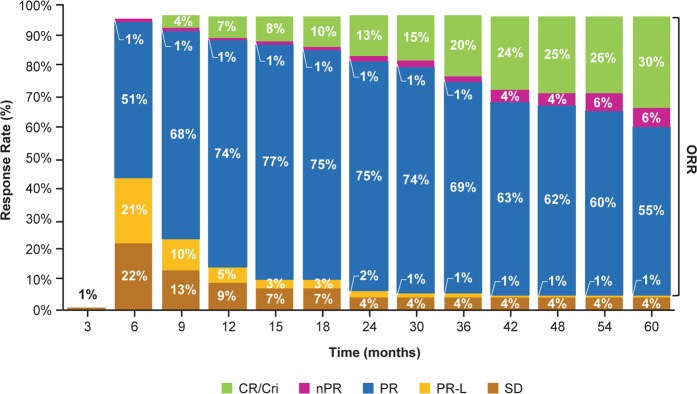
Overall response rate per investigator assessment with first-line ibrutinib. Cumulative best response over time in all patients. Percentages of patients in each category of response may not total the overall proportion with a response because of rounding. CR complete response, CRi complete response with incomplete marrow recovery, nPR nodular partial response, ORR overall response rate, PR partial response, PR-L partial response with lymphocytosis, SD stable disease

With a median follow-up of 5 years (up to 66 months), the ORR including partial response with lymphocytosis was 92% for patients treated with ibrutinib compared with 37% for patients treated with chlorambucil. The proportion of patients with a best response of CR or CR with incomplete marrow recovery (CRi) increased over time (Fig. 4). In the ibrutinib arm, investigator-assessed CR/CRi rates increased from 11% at the primary analysis (median follow-up, 18 months) to 30% after a median follow-up of 5 years. In the ibrutinib arm, ORR and CR rates for patients with high-risk features (i.e., del[11q] or unmutated IGHV) were consistent with the rates seen for all patients treated with ibrutinib.

### Sustained hematologic improvement

**Fig. 5:**
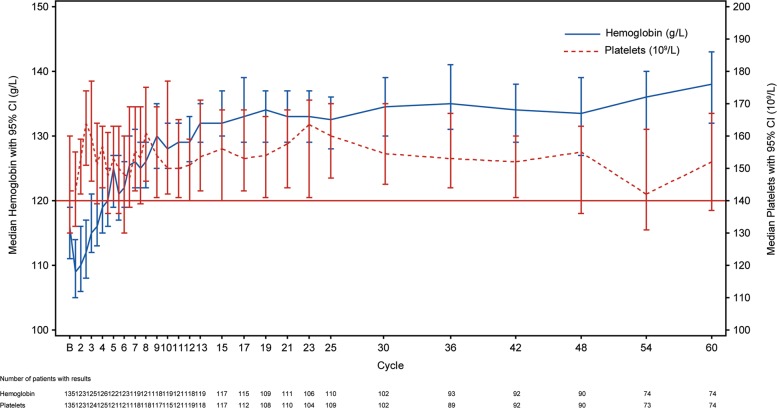
Hemoglobin levels and platelet counts over time in ibrutinib-treated patients. The horizontal line represents the lower limit of normal for platelet counts. CI confidence interval

Among patients with baseline cytopenias treated with ibrutinib, the proportion of patients with sustained hematologic improvement increased over time. Significantly more ibrutinib-treated patients with baseline anemia (hemoglobin ≤11 g/dL) had sustained improvement in hemoglobin levels compared with chlorambucil (90% [46 of 51] vs 45% [25 of 55]; *P* < 0.0001). Similarly, significantly more patients with baseline thrombocytopenia (platelets ≤100 × 10^9^/L) had sustained improvement in platelet counts after ibrutinib treatment compared with chlorambucil (89% [31 of 35] vs 46% [13 of 28]; *P* = 0.0007). For patients treated with ibrutinib, median hemoglobin level was 11.6 g/dL at treatment initiation, 13.2 g/dL at year 1, and 13.7 g/dL at year 5 (Fig. 5). Median platelet counts for patients treated with ibrutinib were 143 × 10^9^/L at treatment initiation, 155 × 10^9^/L at year 1, and 147 × 10^9^/L at year 5 (Fig. 5).

### Patient-reported outcomes and disease-related symptoms

Patient-reported outcomes as assessed with the EQ-5D-5L and FACIT-F were improved with ibrutinib versus chlorambucil. Clinically meaningful improvement in the EQ-5D-5L UIS (≥0.084) [15] and in EQ-5D-5L VAS were observed significantly more frequently with ibrutinib than with chlorambucil (60% vs 44% of patients, *P* = 0.0089; 65% vs 52%, *P* = 0.0329, respectively), though no significant difference between ibrutinib- and chlorambucil-treated patients was observed for the proportion of patients who had clinically meaningful improvement in FACIT-F (63% vs 53%). By repeated measure analysis, ibrutinib resulted in significantly greater score improvements over time in EQ-5D-5L UIS (*P* = 0.0079), EQ-5D-5L VAS (*P* = 0.0003), and FACIT-F (*P* = 0.0018) (Supplementary Fig. 2). There were no differences in EQ-5D-5L UIS, EQ-5D-5L VAS, or FACIT-F after chlorambucil-treated patients crossed over to ibrutinib following PD compared with before chlorambucil-treated patients crossed over (Supplementary Fig. 3).

Consistent with patient-reported outcomes, when compared with chlorambucil, ibrutinib resulted in higher rates of improvements in disease-related symptoms of weight loss, fatigue, fever, night sweats, anorexia, and abdominal discomfort due to splenomegaly (Supplementary Fig. 4).

### Safety

At data cutoff, the median duration of ibrutinib treatment was 57 months (range, 0.7–66 months) (Table 2) and median relative dose intensity was 98%. The most frequent AEs of any grade with ibrutinib were diarrhea (50%), cough (36%), and fatigue (36%) (Table 3), and the prevalence of many AEs decreased with time on treatment.

**Table 3:** Most frequent adverse events over time in patients treated with first-line ibrutinib

Adverse events, *n* (%)	Ibrutinib *n* = 135^a^					
	0–1 year *n* = 135	1–2 years *n* = 123	2–3 years *n* = 111	3–4 years *n* = 100	4–5 years *n* = 89	Total^d,e^ *n* = 135
Any grade^b^	133 (99)	118 (96)	104 (94)	97 (97)	87 (98)	135 (100)
Diarrhea	57 (42)	11 (9)	13 (12)	8 (8)	8 (9)	67 (50)
Fatigue	38 (28)	27 (22)	21 (19)	19 (19)	18 (20)	48 (36)
Cough	26 (19)	13 (11)	12 (11)	10 (10)	11 (12)	49 (36)
Peripheral edema	23 (17)	17 (14)	13 (12)	12 (12)	11 (12)	37 (27)
Anemia	22 (16)	12 (10)	9 (8)	9 (9)	6 (7)	35 (26)
Nausea	27 (20)	9 (7)	5 (5)	3 (3)	3 (3)	35 (26)
Pyrexia	20 (15)	8 (7)	7 (6)	6 (6)	6 (7)	36 (27)
Arthralgia	19 (14)	13 (11)	10 (9)	6 (6)	13 (15)	35 (26)
Upper respiratory tract infection	17 (13)	8 (7)	10 (9)	10 (10)	7 (8)	35 (26)
Hypertension	16 (12)	12 (10)	15 (14)	16 (16)	17 (19)	31 (23)
Constipation	16 (12)	14 (11)	11 (10)	6 (6)	7 (8)	28 (21)
Vomiting	16 (12)	5 (4)	7 (6)	3 (3)	1 (1)	27 (20)
Grade ≥3^c^	78 (58)	48 (39)	30 (27)	39 (39)	32 (36)	112 (83)
Neutropenia	11 (8)	4 (3)	1 (1)	1 (1)	0 (0)	17 (13)
Pneumonia	7 (5)	3 (2)	3 (3)	4 (4)	2 (2)	16 (12)
Hypertension	6 (4)	2 (2)	0	3 (3)	4 (4)	11 (8)
Anemia	8 (6)	1 (1)	1 (1)	0	0 (0)	10 (7)
Hyponatremia	3 (2)	4 (3)	0	0	1 (1)	8 (6)
Atrial fibrillation	2 (1)	0	4 (4)	1 (1)	0 (0)	7 (5)
Cataract	1 (1)	1 (1)	1 (1)	1 (1)	3 (3)	7 (5)
Diarrhea	5 (4)	0	1 (1)	0	0 (0)	6 (4)

^a^One patient did not receive any doses of ibrutinib

^b^Occurring in ≥20% of patients

^c^Occurring in ≥4% of patients

^d^Median 5 years follow-up

^e^Includes 5–6 year data

Among AEs of particular interest, including those identified during early ibrutinib clinical development, hypertension of any grade occurred in 35 (26%) patients, including 13%, 6%, 6%, 5%, and 7% of patients in years 0–1, 1–2, 2–3, 3–4, and 4–5, respectively (Supplementary Fig. 5). Grade 3 hypertension occurred in 12 (9%) patients, with no grade 4 or 5 events. Atrial fibrillation of any grade occurred at any time in 22 (16%) patients, including 6%, 1%, 6%, 3%, and 4% of patients in years 0–1, 1–2, 2–3, 3–4, and 4–5, respectively (Supplementary Fig. 5). Grade 3 atrial fibrillation occurred in 7 (5%) patients, with no grade 4 or 5 events. Major hemorrhage (grouped terms) events occurred in 15 (11%) patients, including 4%, 3%, 1%, 3%, and 2% of patients in years 0–1, 1–2, 2–3, 3–4, and 4–5, respectively (Supplementary Fig. 5). Grade 3 major hemorrhage occurred in 8 (6%) patients, grade 4 in 1 (1%) patient, and no grade 5 events occurred. Of the patients who experienced grade ≥3 major hemorrhage, 6 (67%) were taking concomitant anticoagulation therapy.

Treatment discontinuations decreased over time on ibrutinib, with 7% of patients discontinuing because of AEs in years 0–1, 6% in years 1–2, 5% in years 2–3, 6% in years 3–4, and 1% in years 4–5 (Supplementary Fig. 6). Thirty-eight patients experienced AEs leading to discontinuation of ibrutinib; those reported in ≥2 patients were atrial fibrillation (*n* = 4), and palpitations, pneumonia, and worsening CLL (*n* = 2 each), in addition to 2 deaths with unknown cause.

The rates of dose reductions due to AEs were similar over time (Supplementary Fig. 6), with rates of 9% for patients in years 0–1, 8% in years 1–2, 6% in years 2–3, 5% in years 3–4, and 7% in years 4–5. Dose reductions due to any-grade AEs occurred in 27 patients; 25 (93%) had improvement or resolution of the AE following dose reduction. At the time of data cutoff, 12/27 remained on ibrutinib and 15/27 had discontinued ibrutinib at any time during follow-up after the dose reduction. Reasons for subsequent discontinuation at any time during follow-up were for any AE (*n* = 9; two patients had dose reduction and then discontinued because of the same AE), withdrawal from the study (*n* = 3), PD (*n* = 2), and physician decision (*n* = 1). AEs leading to dose reduction reported in >1 patient were thrombocytopenia (*n* = 3), and anemia, arthralgia, diarrhea, fatigue, and palpitations (*n* = 2 each). After AE-related dose reductions, ibrutinib dose was successfully re-escalated back to previous dose for ≥2 treatment cycles in six (22%) patients, and re-escalated treatment lasted a median of 589 days (preceding dose reductions lasting a median of 103 days).

Ibrutinib dosing was held for ≥7 consecutive days because of any grade AEs in 70 patients and 60 patients had complete resolution of the AE following dose hold. At the time of data cutoff, 36/70 of these patients remained on ibrutinib and 34/70 had discontinued ibrutinib at any time during follow-up after the dose hold. Reasons for subsequent discontinuation at any time during follow-up included any further AE (*n* = 20), withdrawal from study (*n* = 6), PD (*n* = 5), death (*n* = 2), or physician decision (*n* = 1). Of note, only six patients discontinued due to the same AE after the dose hold (atrial fibrillation, cerebral hemorrhage, cognitive disorder, myelodysplastic syndrome, non–small cell lung cancer, and staphylococcal sepsis). For these 70 patients, the median duration between first dose hold of ibrutinib to study treatment discontinuation or last known date alive for those still on treatment was 48 months (maximum 64+ months). Following dose hold, ibrutinib was restarted at the same dose in 42 patients and at a reduced dose in 22 patients.

At the time of data cutoff, 23 patients randomized to ibrutinib died (8 while on treatment); 4 due to PD (all were aged ≥70 years). One AE of pneumonia was considered possibly related to ibrutinib. There were six patients for whom cause of death was unknown, and of the remaining, there were two infections, three second malignancies, and one each reported as multiorgan failure, heart attack, sudden death, heart failure, sepsis, pulmonary fibrosis, and septic shock (Supplementary Fig. 7).

### Outcomes following ibrutinib discontinuation

Outcomes following discontinuation of first-line ibrutinib treatment are shown in Supplementary Table 2. Median OS following discontinuation was not reached (range, 0–64+ months) in patients who discontinued ibrutinib because of AEs (*n* = 29). Only eight patients discontinued ibrutinib because of PD (including two patients due to Richter’s transformation); of these patients, 50% are still alive or had exited study with no known death at the data cut. The median OS following ibrutinib discontinuation due to PD was 20 months (range, 1+ to 28 months). Median PFS for patients who were in CR/CRi at ibrutinib discontinuation was 56 months (95% CI: 44, NE) compared with 33 months (95% CI: 26, 46) for patients who were not CR/CRi at ibrutinib discontinuation (HR [95% CI]: 0.390 [0.118, 1.285]).

Of patients with available follow-up data after ibrutinib discontinuation, 14 patients received subsequent therapy for CLL, including standard chemoimmunotherapy (FCR, BR, or GC) (*n* = 8), chemotherapy (*n* = 3), and novel agents (*n* = 3). Of nine patients with best overall response to subsequent therapy reported, seven responded, one had stable disease, and one had PD. Eleven of the 14 patients remained alive at last follow-up, two patients withdrew consent, and one patient died.

## Discussion

With long-term follow-up of the RESONATE-2 study, single-agent ibrutinib continues to demonstrate significant and durable clinical benefit in older patients, including those with high-risk prognostic features (*TP53* mutation, del(11q), and/or unmutated IGHV). No new safety signals emerged over the extended treatment duration, and many events decreased over time. This is the longest follow-up report of patients receiving first-line treatment with a BTK inhibitor in a phase 3 study to date.

With up to 66 months of follow-up, the median investigator-assessed PFS per iwCLL criteria was not reached in the ibrutinib arm and was 15 months (95% CI [10–19]) in the chlorambucil arm. The rate of PD during ibrutinib treatment was low; only 8/136 (6%) patients progressed while receiving ibrutinib, implying a low rate of developing the BCR pathway mutations associated with PD. Improvement in PFS with first-line ibrutinib compared with chlorambucil remains durable, as evidenced by an 85% reduction in the risk of progression or death. Accordingly, when comparing similar timepoints between studies, the 3-year PFS rate with ibrutinib (82%) was higher than that with chlorambucil (25%) and appears higher than rates previously reported for first-line chemoimmunotherapy with FCR (70%) or BR (55%) in an older patient population than in the CLL10 study, although the proportion of patients with unmutated IGHV was higher in CLL10 than in the current study [16, 17]. Three-year PFS rates for ibrutinib also appeared favorable compared with chlorambucil in combination with obinutuzumab (<40%) in older, less fit patients in the CLL11 study [5].

In addition, ibrutinib substantially improved PFS in high-risk patients with del(11q) or unmutated IGHV compared with chlorambucil. Whereas the presence of del(11q) [18] or unmutated IGHV [16, 19] confers poor outcomes in the chemoimmunotherapy setting, we observed prolonged PFS with ibrutinib compared with chlorambucil in patients with del(11q) or with unmutated IGHV. Patients with the composite high prognostic risk genomics of del(11q), unmutated IGHV, or *TP53* mutation experienced markedly improved PFS with ibrutinib, with a 92% reduction in risk of PD or death versus chlorambucil. However, there were no meaningful differences in PFS for ibrutinib-treated patients when these high-risk factors were evaluated individually (del[11q], unmutated IGHV, or *TP53* mutation). The exclusion of patients with del(17p) is an important limitation of this analysis given its prognostic significance and frequent overlap with *TP53* mutations [20]. Taken together, our results suggest that these high-risk prognostic features may not have meaningful prognostic value with ibrutinib-mediated inhibition of the BCR pathway, unlike with chemoimmunotherapy.

Cross-study analyses suggest that the clinical benefit is highest when ibrutinib is used as initial therapy versus as a later line of therapy [7], and the present study demonstrates the durability of those outcomes in the first-line setting with more than 5 years follow-up. Real-world studies of patients receiving first-line treatment, including patients who would have been excluded from RESONATE-2 due to age <65 years or the presence of del(17p), demonstrate similar response rates (71–82% vs 92%) and proportion of discontinuations due to AEs (51–63% vs 52% of patients who discontinued) as the present study [21–24].

Additional randomized trials have found that adding rituximab to ibrutinib does not increase PFS compared with single-agent ibrutinib in the first-line setting [25] and in relapsed/refractory patients or high-risk patients with del(17p) or *TP53* mutations receiving first-line treatment [26]. In the iLLUMINATE study of ibrutinib plus obinutuzumab, median PFS was also not reached for patients with del(11q) nor for patients with unmutated IGHV, similar to what we report here for single-agent ibrutinib (Fig. 3). These results with del(11q) and IGHV mutational status are also consistent across two other phase 3 studies of ibrutinib in different patient populations and as a single agent or in combination regimens [6, 18, 27]. A pooled analysis of three phase 3 randomized studies (RESONATE, RESONATE-2, and HELIOS) further showed that the prognostic risk factors of del(11q) and unmutated IGHV traditionally associated with worse outcomes in patients with CLL have less prognostic significance with ibrutinib therapy in patients without del(17p) [28].

Our results also demonstrate an improved depth of response over time with first-line ibrutinib. Investigator-assessed CR/CRi rates in ibrutinib-treated patients improved from 11% at the primary analysis (median follow-up 18.4 months) [8] to 30% after a median of 5 years follow-up. In addition, we continue to observe sustained improvement in anemia and thrombocytopenia with ibrutinib, which are important and frequent reasons patients with CLL initiate treatment. These improvements may help alleviate the fatigue that is a major component of symptom burden and reduced quality of life in patients with CLL [29], especially those with advanced age and multiple comorbidities [29]. Given that patients with CLL may remain on ibrutinib for many years, durable improvement in quality of life during treatment is an important goal. In this study, ibrutinib treatment improved patient-reported outcomes and disease-related symptoms that were sustained through extended follow-up, in contrast to the worsened quality of life outcomes reported by patients treated with chlorambucil, as PD occurred earlier and more frequently.

As the majority of patients with CLL (including those in this study) are elderly and may be less tolerant of toxicities, treatments with a tolerable safety profile in long-term use are essential. Late high-grade toxicities have been observed with other CLL therapies [30]. In this study, no unexpected AEs were identified after extended follow-up of ibrutinib-treated patients. Patients continued to experience new AEs throughout extended treatment, with some AEs appearing as late events, such as cataracts, fall, and herpes zoster, although the impact of aging in this patient population with extended follow-up cannot be fully accounted for. In the primary analysis of RESONATE-2 (median follow-up, 18 months), the three most common AEs were diarrhea (42%), fatigue (30%), and cough (22%) [8], while in the current analysis at median of 5 years of follow-up (~3.3-fold longer exposure to ibrutinib) these AEs continue to be the most common events in 50%, 36%, and 36% of patients, respectively. Compared with a younger enrolled patient population treated with ibrutinib plus rituximab for a shorter median follow-up of 33 months in the phase 3 ECOG1912 study, more ibrutinib-treated patients in RESONATE-2 (~1.8-fold longer exposure to ibrutinib) experienced grade ≥3 AEs (83% vs 58%) overall, although there was a lower frequency of grade ≥3 AEs in the ibrutinib plus rituximab cohort compared with the FCR cohort in ECOG1912 (58% vs 72%) [31]. Compared with a similar patient population in the ALLIANCE study, fewer ibrutinib-treated patients in RESONATE-2 experienced grade ≥3 hypertension than ibrutinib-treated patients in the ALLIANCE study (8% vs 29%), despite the difference in follow-up (median: 60 months vs 38 months, respectively) [25].

Overall, many AEs decreased over time in this study, with some exceptions, such as hypertension (the prevalence of grade ≥3 hypertension remained stable over time). The incidence of major hemorrhage was generally highest in the first 2 years of treatment and decreased thereafter. Based on prior reports, the risk for bleeding with ibrutinib is most often observed within the first 12 months of treatment and then decreases over time [27, 32, 33]. Similarly, atrial fibrillation typically occurs early after ibrutinib initiation and remains constant or declines over time [7, 34–36]. In this study after a median follow-up of 5 years, the cumulative rate of major hemorrhage increased from 4% at the primary analysis to 11%, and for atrial fibrillation increased from 6% to 16%; however, few patients required dose reduction or discontinued because of these AEs. Overall, dose reductions and discontinuations due to AEs were infrequent and discontinuations due to AEs decreased over time with continued treatment, with 73% of patients receiving ibrutinib for >3 years. The ongoing incidence of new AEs and increasing prevalence of hypertension highlights the importance of ongoing follow-up and monitoring during treatment to maximize optimal management of AEs with dose modifications (dose holds and reductions) and thus mitigate the impact of AEs and enable patients to continue to benefit from ongoing first-line ibrutinib.

As novel agents continue to be developed for CLL, long-term data are crucial to inform practice. Additional BTK inhibitors in development for CLL have shown encouraging efficacy, but results of randomized comparative studies are not yet available and these agents lack long-term safety and efficacy data [37, 38]. Here, we demonstrated that with a median of 5 years of follow-up, over half of patients with CLL/SLL were able to receive long-term continuous first-line treatment with single-agent ibrutinib and had sustained efficacy benefits (70% of ibrutinib-treated patients estimated progression-free), including—importantly—in patients with high-risk prognostic features, such as del(11q) or unmutated IGHV.

## Supplementary information

Supplemental Material
