# Supplementary material for: Long-term efficacy and safety of first-line ibrutinib treatment for patients with CLL/SLL: 5 years of follow-up from the phase 3 RESONATE-2 study
Source: Leukemia. 2019 Oct 18;34(3):787–98. doi: 10.1038/s41375-019-0602-x (PMC7214263; doi:10.1038/s41375-019-0602-x)
Supplement: Supplementary file 1 — Supplemental Material [file 41375_2019_602_MOESM1_ESM.docx]

**Supplementary Information**

**Supplementary Table 1.** Overall survival adjusted for crossover from chlorambucil to ibrutinib after disease progression

| **Method** | **Cox model** | |
| --- | --- | --- |
|  | **HR** | **95% CI** |
| ITT (Not censored at crossover)^a^ | 0.450 | 0.266–0.761 |
| RPSFT model^b^ | 0.300 | 0.171–0.525 |
| Censored at crossover^a^ | 0.635 | 0.334–1.207 |

*CI* confidence interval, *ECOG PS* Eastern Cooperative Oncology Group performance status, *HR* hazard ratio, *ITT* intention-to-treat, *OS* overall survival, *RPSFT* rank-preserving structural failure time

^a^Analysis stratified by two randomization factors: ECOG PS (0/1 vs 2) and Rai stage (0/I/II vs III/IV) at baseline as reported in the interactive web response system

^b^Cox model including treatment and baseline covariates to compensate for any lack of balance between treatment arms and improve precision (ECOG PS, Rai stage, age, sex, bulky disease, del[11q], region, ethnicity, lactate dehydrogenase, β2-microglobulin, creatinine clearance)

**Supplementary Table 2.** Outcomes following first-line ibrutinib discontinuation

|  | **Discontinued due to AEs**  ***n*=29** | **Discontinued due to PD**  ***n*=8** | **Discontinued due to any cause other than death**  ***n*=48** |
| --- | --- | --- | --- |
| Median (95% CI) time on study after discontinuation, months | 21 (12–43) | 20 (4–28) | 14 (9–21) |
| Median (95% CI) OS after discontinuation, months | NE (16–NE) | 20 (1–NE) | NE (16–NE) |
| Remaining alive,^a^ *n* (%) | 20 (69) | 4 (50) | 33 (69) |

*AEs* adverse events, *CI* confidence interval, *NE* not estimable, *NR* not reached, *OS* overall survival, *PD* progressive disease

^a^Includes patients who exited the study with no known death

+: indicates censored observation

**Supplementary Methods**

*Assessments*

Sustained hematologic improvement was protocol-specified and defined as hematologic improvement sustained continuously for ≥56 days without blood transfusion or growth factors: platelet counts >100 × 10^9^/L if baseline ≤100 × 10^9^/L or increase ≥50% over baseline; hemoglobin >11 g/dL if baseline ≤11 g/dL or increase ≥2 g/dL over baseline. Patient-reported outcomes included Functional Assessment of Chronic Illness Therapy-Fatigue (FACIT-F) scale score^1^, EuroQol five dimension, five level (EQ-5D-5L) Visual Analog Scale (VAS) score^2^, and EQ-5D-5L utility index score (UIS) (^©^EuroQol Research Foundation. EQ-5D™ is a trademark of the EuroQol Research Foundation)^2^. Major hemorrhage was defined as any hemorrhagic event grade ≥3 in severity or resulting in one of the following: intraocular bleeding causing vision loss, transfusion of ≥2 units of red cells or equivalent amount of whole blood, hospitalization, or prolongation of hospitalization. Outcomes of the composite high-prognostic risk group that include *TP53* mutation status were non-prespecified post-hoc analyses.

*Statistical analysis*

Hazard ratios were calculated using Cox regression models and treatment arms were compared using a two-sided log-rank test stratified by randomization factors (Eastern Cooperative Oncology Group performance status and Rai stage). Hematologic improvement was analyzed by Fisher’s exact test. Patient-reported outcomes were evaluated based on the proportion of patients who had a clinically meaningful change in score from baseline, defined as ≥3 points for FACIT-F, ≥7 points for EQ-5D-5L VAS, and ≥0.08 points for EQ-5D-5L UIS, as well as mixed-model repeated-measures analysis.

References

1. Yellen SB, Cella DF, Webster K, Blendowski C, Kaplan E. Measuring fatigue and other anemia-related symptoms with the Functional Assessment of Cancer Therapy (FACT) measurement system. *J Pain Symptom Manage.* 1997; **13**: 63–74.

2. EuroQol Group. EuroQol--a new facility for the measurement of health-related quality of life. *Health Policy*. 1990; 1**6**: 199–208.

**Supplementary Figure 1.** Participant flow diagram


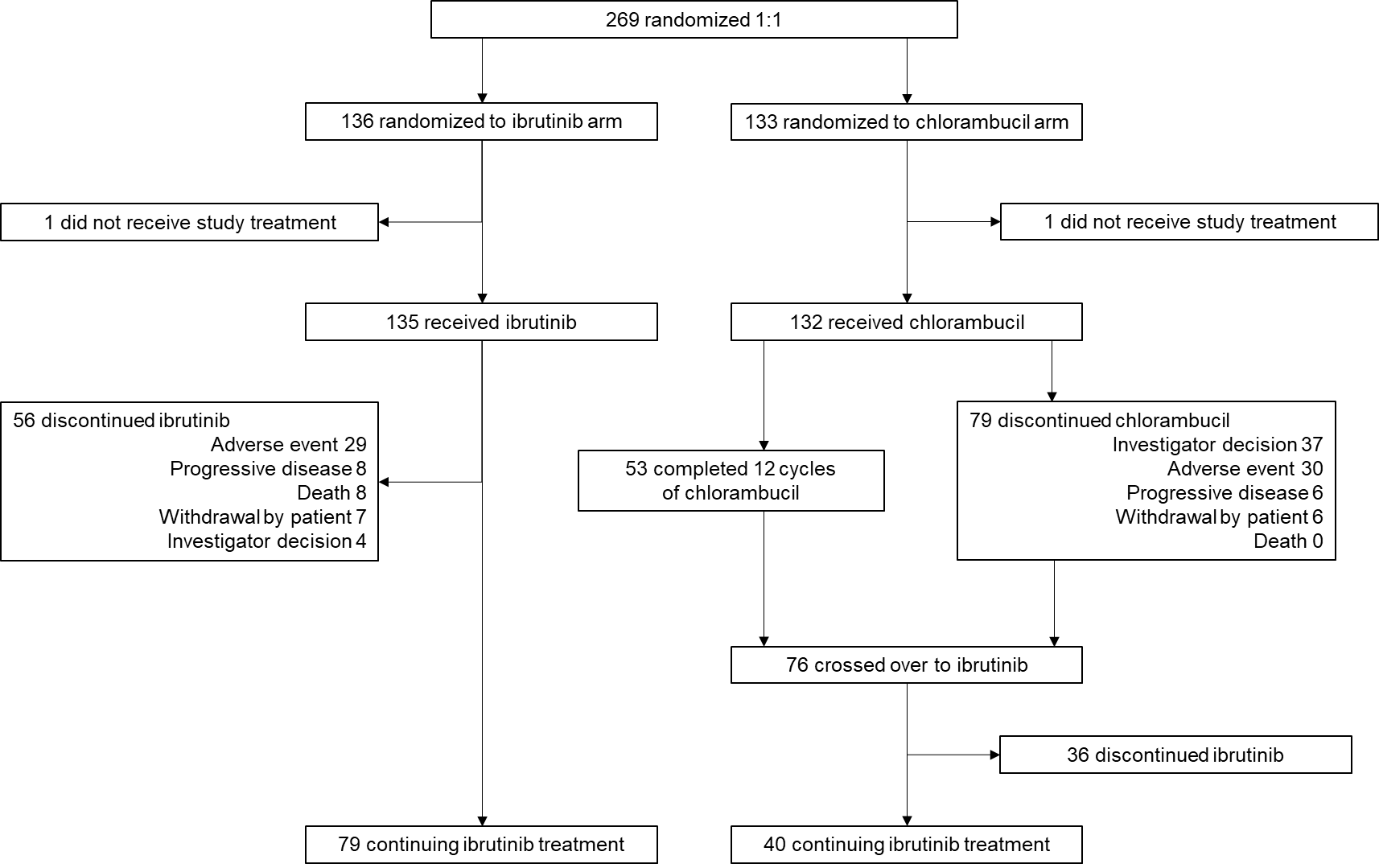


**Supplementary Figure 2.** Least squares mean change from baseline at each visit by mixed model repeated measures analysis for (A) FACIT-F, (B) EQ-5D-5D VAS, and (C) EQ-5D-5L UIS


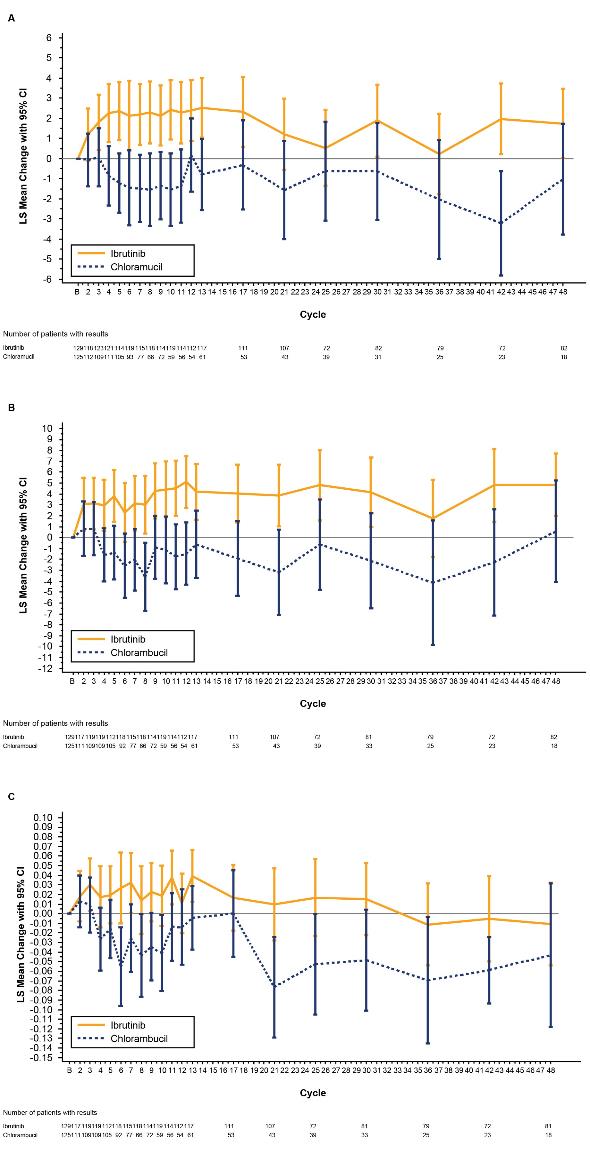


*CI* confidence interval, *EQ-5D-5L UIS* EQ-5D-5L utility index score, *EQ-5D-5L VAS* EQ-5D-5L Visual Analog Scale score, *FACIT-F* Functional Assessment of Chronic Illness Therapy–Fatigue

**Supplementary Figure 3.** Mean change from baseline^a^ at each visit before and after crossover from chlorambucil to ibrutinib for (A) FACIT-F, and (B) EQ-5D-5D VAS, and (C) EQ-5D-5L UIS


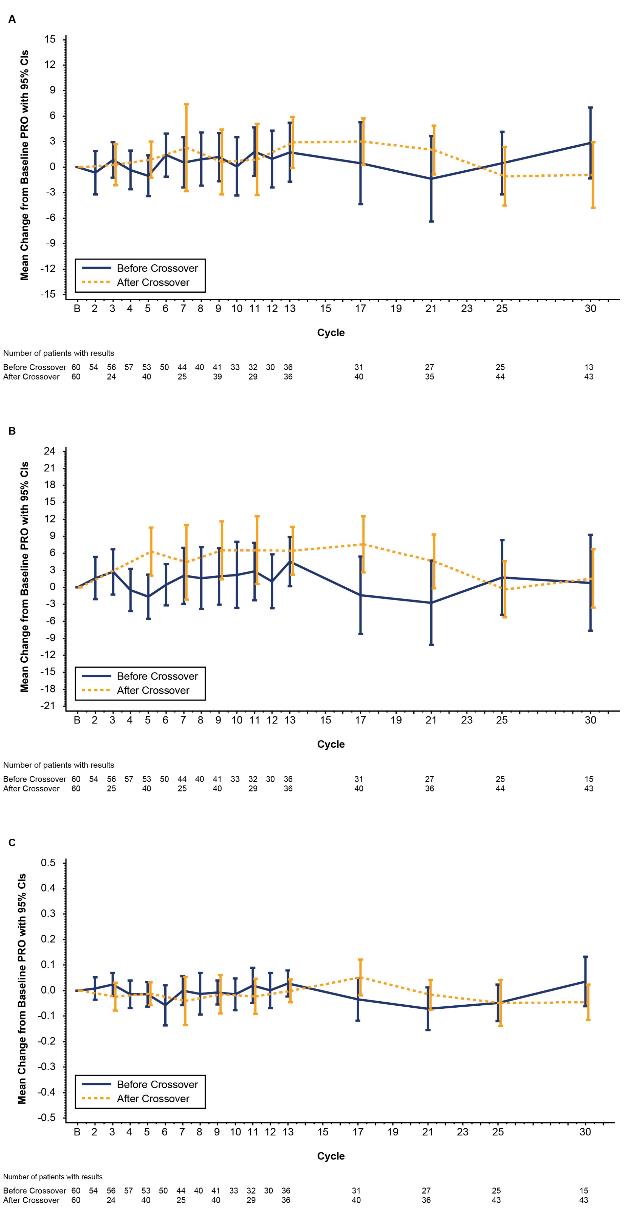


*CI* confidence interval, *EQ-5D-5L UIS* EQ-5D-5L utility index score, *EQ-5D-5L VAS* EQ-5D-5L Visual Analog Scale score, *FACIT-F* Functional Assessment of Chronic Illness Therapy–Fatigue, *PRO* patient-reported outcome

^a^For PRO data after crossover from chlorambucil to ibrutinib, baseline is defined as the last non-missing visit on or prior to crossover. For cycle number after crossover, cycle 1, day 1 is defined as the date of the first dose of ibrutinib.

**Supplementary Figure 4.** Improvement in disease-related symptoms^a^

^a^Defined as a change of at least 1 grade from baseline for at least 2 consecutive assessments at any time, as assessed by the investigator, among patients with symptoms of grade ≥1 at baseline

**Supplementary Figure 5.** Percentage of patients with adverse events of interest over time with first-line ibrutinib

**Supplementary Figure 6.** Dose reduction and discontinuation rates due to adverse events over time with first-line ibrutinib


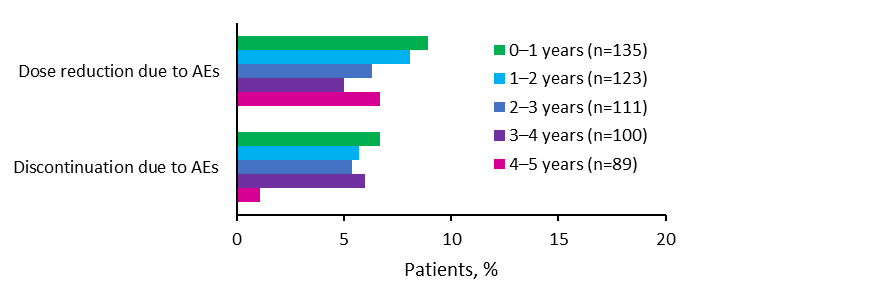


*AEs* adverse events

**Supplementary Figure 7.** Overall survival by cause of death and age at enrollment in patients randomized to first-line ibrutinib who died during the study^a^


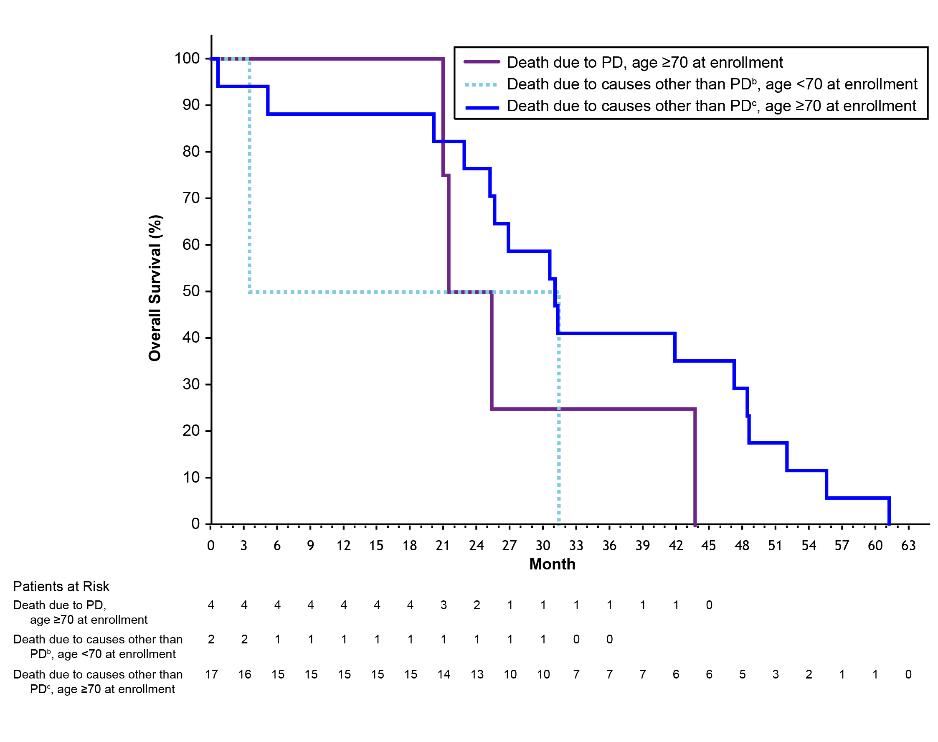


*PD* progressive disease

^a^There were no deaths due to PD in patients <70 years

^b^The causes of death were unknown (n=1), and Klebsiella pneumonia infection (n=1).

^c^The causes of death were unknown (n=5), secondary malignancy (n=3), multi-organ failure (n=1), heart attack (n=1), pneumonia (n=1), sudden death (n=1), infection (n=1), sepsis (n=1), pulmonary fibrosis (n=1), heart failure (n=1), septic shock (n=1).
